# Returning home during the pandemic: a thematic analysis describing experiences of people with substance use disorders released early from New Jersey prisons during COVID-19

**DOI:** 10.1186/s40352-023-00208-x

**Published:** 2023-02-27

**Authors:** Madeline H. Bono, Peter Treitler, Brendan Saloner, Stephen Crystal

**Affiliations:** 1 Graduate School of Applied and Professional Psychology, Rutgers, The State University of New Jersey, 152 Frelinghuysen Road, Piscataway, NJ 08854 USA; 2 Center for Health Services Research, Institute for Health, Health Care Policy and Aging Research, Rutgers, the State University of New Jersey, New Brunswick, NJ USA; 3 Department of Health Policy and Management, Johns Hopkins University, Bloomberg School of Public Health, Baltimore, MD USA

**Keywords:** Substance Use Disorder, Opioid Use Disorder, Decarceration, COVID-19, Reentry Services, Qualitative Research, Prisons

## Abstract

**Background & aims:**

The COVID-19 pandemic created intersecting health risks for incarcerated people with a history of substance use disorder (SUD). To reduce exposure to COVID-19 in prison, several US states enacted decarceration legislation. New Jersey enacted the Public Health Emergency Credit Act (PHECA), granting early release to thousands of incarcerated persons meeting eligibility criteria. This study undertook to explore how large scale decarceration during the pandemic impacted the reentry process for released individuals with SUDs.

**Methods:**

Twenty seven participants involved in PHECA releases – 21 persons released from NJ carceral facilities with past/present SUDs (14 with opioid use disorder, 7 with other SUDs) and 6 reentry service providers acting as key informants – completed phone interviews on PHECA experiences from February–June 2021. Cross-case thematic analysis of transcripts identified common themes and divergent perspectives.

**Results:**

Respondents described challenges consistent with long-documented reentry difficulties including housing and food insecurity, difficulty accessing community services, insufficient employment opportunities, and limited access to transportation. Challenges that were pertinent to mass release during a pandemic included limited access to communication technology and community providers and community providers exceeding enrollment capacity. Despite reentry difficulties, respondents identified many areas where prisons and reentry service providers adapted to meet novel challenges presented by mass decarceration during the COVID-19 pandemic. Facilitators made available by prison and reentry provider staff included providing released persons with cell phones, transportation assistance at transit hubs, prescription support for medications for opioid use disorder, and pre-release assistance with ID and benefits through NJ’s Joint Comprehensive Assessment Plan.

**Conclusions:**

Formerly incarcerated people with SUDs experienced reentry challenges during PHECA releases similar to those that occur during ordinary circumstances. Despite barriers faced during typical releases and novel challenges unique to mass release during a pandemic, providers made adaptations to support released persons' successful reentry. Recommendations are made based on areas of need identified in interviews, including reentry service provision facilitating housing and food security, employment, medical services, technology fluency, and transportation. In anticipation of future large scale releases, providers will benefit from planning ahead and adapting to address temporary increases in resource demands.

**Supplementary Information:**

The online version contains supplementary material available at 10.1186/s40352-023-00208-x.

## Introduction

Incarcerated individuals with substance use disorders (SUDs) are uniquely vulnerable to COVID-19 (Melamed et al., 2020; Mukherjee & El-Bassel, 2020). Overcrowding, dormitory-style housing, inadequate ventilation, and limited space for quarantining contributed to COVID-19’s rapid spread through correctional facilities (Heard, 2021; Leibowitz et al., 2021; Reinhart & Chen, 2021; Vest et al., 2021), with incarcerated people facing COVID-19 mortality rates twice that of the non-incarcerated population (National Commission of COVID-19 and Criminal Justice, 2020). For individuals with SUDs – estimated to comprise over 60% of the incarcerated US population (Bronson et al., 2017) – the risk of negative outcome following COVID-19 infection is also substantially elevated (Baillargeon et al., 2021; Wang et al., 2021).

The overrepresentation of individuals with SUDs in the US’s carceral system is just one of myriad structural inequities perpetuated by the criminal legal system (American Public Health Association, 2020; Wang et al., 2020). In the US, individuals from marginalized racial and ethnic groups are more likely to be detained following prosecutable offenses, and receive disproportionately punitive legal outcomes, which has contributed to a disproportionately high representation of Latino/a and Black individuals in the prison system, when compared with the general US population (American Public Health Association, 2020; Brinkley-Rubinstein & Cloud, 2020; Kutateladze et al., 2014). In public health spheres, mass decarceration has been identified as a central means of countering the criminal legal system’s structural inequities (American Public Health Association, 2020).

In light of COVID-19’s adverse effects on incarcerated people, and intersecting disparities in COVID-19 health outcomes for marginalized racial and ethnic groups and people with SUDs, implementing large scale decarceration can have important public health implications during the pandemic (Baillargeon et al., 2021; Barnert et al., 2021; Klein et al., 2021; Macmadu et al., 2020; Nowotny et al., 2021). Following the pandemic’s onset, several jurisdictions enacted emergency decarceration efforts to reduce COVID-19 infection rates in carceral facilities (Franco-Paredes et al., 2020; Heard, 2021). One such effort was New Jersey’s Public Health Emergency Credit Act (PHECA), which granted certain prisoners an up-to-eight-month sentence reduction (Parmely, 2021); through this legislation, NJ conducted one of the largest-ever rapid reductions of a state prison population (Sinha, 2021).

After release, individuals with SUDs are especially vulnerable. These individuals are more likely than other released individuals to contract infectious diseases (Butler et al., 2011), to incur fatal and nonfatal overdoses (Crystal et al., 2021; Green et al., 2018; Mital et al., 2020), and to recidivate (Dowden & Brown, 2002; Link & Hamilton, 2017). The intersection of risk factors among released individuals – for example individuals having both SUD and membership to one or more structurally marginalized groups (Lockwood et al., 2015) – leaves many released individuals facing compound barriers to reentry. Reentry literature has thus emphasized the importance of ensuring that released individuals have access to housing, employment, medical care, and SUD services (i.e. counseling and medication for opioid use disorder [MOUD]), both while incarcerated and post-release (Harding et al., 2019; Hlavka et al., 2015; Visher et al., 2017). Unfortunately, speaking to the far reach of the criminal legal system’s structural inequities, barriers to released individuals accessing each of these necessities under typical release circumstances have been long-documented (Doyle et al., 2022; Harding et al., 2019; Stopka et al., 2022).

With PHECA facilitating prison releases on an unprecedented scale, during the public health emergency, there were initially concerns 1) that some of these documented barriers to reentry might be amplified; and 2) that this legislation might impact public safety. Preliminary investigations on recidivism rates for PHECA-released individuals reentering the community indicated that PHECA releases did not elevate public safety risks (Yi, 2022a, 2022b), but as-of-yet, the lived experiences of PHECA-released individuals are not represented in the literature. This is a crucial opportunity to explore the first of these concerns: what were the unique experiences of individuals released through rapid large scale decarceration efforts during COVID-19, particularly those at high-risk of negative post-release outcomes, such as individuals with SUDs? In anticipation of large scale releases, guidelines for successful reentry during the pandemic were published, recommending for released individuals to receive standard recommended reentry services and increased responsivity among providers in adapting to COVID-19 circumstances (e.g. providing technology-based services) – but follow-up studies to examine the success of these efforts are now needed (National Academies of Sciences, Engineering, and Medicine, 2020; Howell et al., 2020; Desai et al., 2021).

Examining the experiences of individuals with SUDs released through PHECA legislation can inform person-centered planning for future large scale releases. To this aim, this study reports on a rapid-cycle qualitative evaluation of PHECA, exploring the experiences of individuals with SUDs and reentry service providers who worked with them, and investigating the following research questions:

- R1: What were the most pressing needs facing individuals with SUDs released during the pandemic, and to what extent were these addressed by existing service systems?
- R2: Were individuals with SUDs prepared for release, and what additional services/activities could have better prepared them?
- R3: What impact did PHECA legislation have on service providers’ capacity to meet the needs of released individuals with SUDs?
- R4: What lessons can be learned from NJ’s mass decarceration efforts and how can they inform future decarceration events?

## Methods

### Participants

Two distinct participant groups were recruited. Releasee participants were individuals with SUDs released early through PHECA from NJ state prisons during COVID-19. Eligibility criteria included being 18 + , self-reported past/present SUD, and early release through PHECA. Additional service provider participants – senior staff representatives from NJ reentry service organizations – were recruited as key informants (Payne & Payne, 2004), so that their perspectives as specialists in the reentry service system could be incorporated and maximize contextual understanding of research questions. Participants were recruited until thematic saturation was attained (Sandelowski, 1995). Respondent characteristics are presented in Table 1.

**Table 1:** Respondent Characteristics (*N* = 27)

	**N**	**Median**/Percentage^a^
Releasee Respondents	21	77.8%
Median Age (Range)	21	**44.7**(31–60) years^b^
Sex		
Female	2	9.5%
Male	19	90.5%
Race/ethnicity		
Non-Hispanic White	8	38.1%
Non-Hispanic Black	10	47.6%
Hispanic	2	9.5%
Native American	1	4.8%
Service Provider Respondents	6	22.2%

^a^Demographic details for service provider respondents are not provided, in efforts to maintain anonymity, given the close-knit nature of community service provision. Only “Releasee Respondents” and “Service Provider Respondents” percentages are calculated in terms of total participant pool. All releasee respondent demographics percentages are calculated in terms of total releasee respondent pool

^b^1 releasee respondent declined to share their age

#### Releasee participants

Releasee participants were 21 individuals with SUDs, 14 of whom specified their SUD as opioid use disorder (OUD) and 7 of whom had other SUDs. Participants were recruited via convenience sampling. Volunteers of America of Delaware Valley (VOA) – a reentry organization serving southern NJ counties – distributed study flyers to individuals involved with their organization. Study staff were provided with contact information of individuals who expressed interest.

#### Provider participants

Six provider respondents were recruited using purposive sampling. Study staff identified service providers involved in NJ reentry organizations through professional networks and internet searches and invited them to complete interviews. Respondents included senior staff representatives from: four agencies that exclusively provide reentry services; one homeless shelter; and one county social services office. Three of the providers worked at 501(c)3 nonprofits and three worked at state-funded organizations. Service provider demographics are excluded from Table 1 due to the small number of interviewees.

### Interviews

Three masters-level interviewers conducted semi-structured telephone interviews with participants between February and June 2021.

Separate interview guides were developed for released individuals and reentry service providers (See Additional files 1 & 2). Interview topics for released persons queried: 1) incarceration experiences during the pandemic; 2) release preparation; 3) SUD treatment experiences; 4) availability and utilization of services in the community; and 5) challenges and unmet needs. Reentry service provider interview topics queried: 1) provider organizations’ preparations for mass release following the PHECA’s passage; 2) inter-organizational collaboration in preparation for large scale release; 3) providers’ perspectives on released persons’ preparedness for reentry; and 4) providers’ sense of reentry programming availability during PHECA. Interviews were audio-recorded and transcribed. They averaged ~ 50 min in length. Each releasee participant was issued a $50 gift card for their participation.

### Coding and analysis

Two research team members experienced in qualitative methods co-created separate codebooks to guide analyses of released individual and service provider transcripts respectively. Initial codebooks were developed based on the interview guides’ thematic elements, and additional codes were added inductively based on transcript reviews. Researchers independently coded each full interview transcript using Dedoose software and met weekly to review and resolve any discrepancies between codes. Final codes were applied based on mutual consensus. Coded data were then analyzed using cross-case thematic analysis (Braun & Clarke, 2006).

## Results

Emergent themes from 27 interviews are presented, with themes detailed in sections based on the chronological sequence of PHECA legislation enactment. Quotes from released persons are attributed in format *“(R####)”* and service providers in format *“(P###)”.*

### Learning about PHECA, prior to legislation enactment

Although not all releasee respondents detailed whether or not they learned about PHECA in advance of release, those who did relayed that there was lack of clarity in communications about PHECA; persons’ eligibility for early release; and/or PHECA procedures, resulting in significant uncertainty regarding the effect of legislation on release dates. One released individual detailed *“I didn't even know that I was going to fall inline with that law [PHECA]. So when that law happened, it's like they started kicking people out. Pretty much, you just know that day they're going to release you.” (R2114).* All releasee respondents who described their pre-release understanding of PHECA procedures endorsed feeling that PHECA-related communications were ineffective in conveying all of the necessary information. One releasee respondent described *“In the period of like two months, I was given like five different release dates. How the hell are you supposed to make any plans when your release date is changing and they think it's not a big deal.” (R2115).* Released individuals had idiosyncratic explanations for the limited extent of PHECA-related communications, which spanned delays in the legislation passing, speculations that prison staff were withholding information, and information being circulated through rumor.

Provider respondents also reported challenges in gaining clarity around PHECA procedures. Most providers reported minimal coordination between their agencies and NJDOC prior to the initial PHECA release on November 4^th^, 2020. As one provider detailed: *“There was really no information on what the process was going to be. We just knew it was going to happen.” (P155)*. One provider reported receiving release details directly from NJDOC, and – although NJDOC supplied this provider’s organization with a preliminary estimation of release numbers – the data was reportedly not sufficiently detailed for the provider’s organization to contact individuals and initiate release preparation activities.

Some providers detailed other avenues through which they sought out information about PHECA, with different providers sourcing information from the news, internet, and other agencies. Several providers reported being given access to a list of upcoming PHECA releases through other involved county agencies (e.g., Department of Human Services [DHS], governor’s office). Respondents conveyed appreciation for information receipt when the information proved correct and/or when it contributed to preparation and planning for PHECA.

### Pre-release services, in preparation for PHECA release

In ordinary circumstances, people in NJ prisons can opt into pre-release programming to enhance their preparedness for reentry, including vocational/education training and SUD services (i.e., AA [Alcoholics Anonymous]/NA [Narcotics Anonymous], MOUD). Given the public health emergency, many COVID-related changes to prison programming were already in effect when PHECA was enacted. Several released individuals described that COVID-related suspensions of vocational/educational services disrupted their feelings of preparedness. As one releasee respondent described: “*I was planning on being there with those certificates, or those trades… I had made a lot of plans based off of that… so then when I didn't have that… I had to kind of change everything around.” (R2107).* Despite that several interviewees identified changes to/suspension of pre-release services to be challenging, respondents also detailed instances where service providers successfully adapted to address these challenges, including examples such as: DOC social workers facilitating phone intakes with outside organizations prior to release – when previously these services would have been able to be in person; and plans to establish virtual programming for incarcerated individuals.

Releasee respondents also described that there were COVID-19-related suspensions/disruptions to AA/NA services while incarcerated, although no respondents were explicit about how this impacted their readiness for release. Of the 21 released persons interviewed, the 14 who self-reported their specific SUD to be an opioid use disorder (OUD) described having been offered MOUD services (i.e. buprenorphine, naltrexone, and/or methadone) by prison staff. Nine of the 14 respondents with OUD initiated MOUD during their incarceration. These individuals receiving MOUD reported that their reentry planning for releases through PHECA comprised prison staff establishing appointments with community MOUD providers and Intensive Recovery Treatment Support (IRTS), a program specializing in reentry coordination for released individuals with SUDs (Swarbrick et al., 2019). One interviewee described their satisfaction with prison staff’s coordination of post-release SUD-care: *“Through the prison, through social services [post-release SUD/MOUD services were arranged]… they set you up with the interview for your aftercare, what doctor, what place you want to go to for continued treatment. So they set all that up for me even during COVID. So they made sure all that stuff got done.” (R2125).*

Per NJDOC policy, all incarcerated individuals complete NJDOC’s Joint Comprehensive Assessment Plan (J-CAP) prior to release with prison staff assistance. J-CAP comprises applications for Medicaid, welfare, and SNAP, and receiving official state ID from the Motor Vehicles Commission. One released individual described of J-CAP services *“It seems like a couple of the social workers really … I feel they really kind of went out of their way the last couple of weeks trying to get stuff ready for me.” (R2115).* This reflects a sentiment shared by many released individuals, the majority of whom described J-CAP procedures as helpful.

When respondents expressed dissatisfaction with J-CAP procedures, two themes were identified. One theme, endorsed most frequently amongst respondents dissatisfied with reentry planning, was that J-CAP procedures were disrupted for PHECA releasees. Reported disruptions included individuals being released without official government ID and benefit applications being misfiled (e.g., sent to the wrong county; benefit packets returned to NJDOC). One releasee respondent spoke to their experience of J-CAP disruptions: *“We did… reentry stuff, telling us what we had to sign up for to get a social security card, birth certificate, state ID. I didn’t get the state ID… though I was eligible for it, but I did get my birth certificate and social.” (R2108).*

The second theme amongst individuals dissatisfied with pre-release planning was that released persons were not given sufficient information to ensure 1) that they understood their reentry planning or 2) that they knew how to access enrolled-in benefits post-release.

As one released individual expressed “*I didn’t even read [reentry plan documentation] that well, man, because it didn’t make no sense.” (R2160).* Although this was less frequently endorsed as a source of frustration than disruptions to standard J-CAP procedures, individuals who did report challenges understanding J-CAP reported extreme dissatisfaction with reentry planning for their release.

### Mass release on November 4^th^

Eleven of the 21 releasee respondents were released on November 4^th^, 2020. This was the first day that PHECA-eligible individuals were released and was the largest-ever single-day prison release in the US, with 2,258 people returning to the community (Sinha, 2021). A common theme in released person and provider interviews were descriptions of the unique circumstances of the November 4^th^ mass release.

*“There was so many people that got released that they couldn't drop off 3000 people, all at [Transit Hub A] or [Transit Hub B]. They had to break it up 100 go here, 100 go there. And different times that we would leave. It'd be a crew set to leave at 8:00. Then four hours later, there'd be another crew set to leave at 4:00.” (R2195)* one released person described. Perhaps reflecting inconsistency in peoples’ experiences on November 4^th^, interviewees described different details about the November 4^th^ mass release. Some differences in details could be attributed to interviewees being released persons versus providers, and differences in release location and time of day.

Several providers conveyed that NJDOC had notified organizations that released persons would be dropped-off at major NJ transit hubs over the course of the day, with drop-offs continuing until late in the evening. One provider relayed that brief prison lockdowns occurred over the day and contributed to delays in transport. Several provider organizations arranged to have staff present at major transit hubs to meet released individuals, engaging in brief needs assessments and service connection. At transit hubs, reentry service providers offered released individuals supplies such as food, water, and backpacks; allowed released persons to use providers’ cell phones to speak with personal contacts; provided transportation via organization vans equipped with PPE; and connected released persons to community reentry service organizations. Many released persons expressed that financial constraints prevented them from using public transportation at transit hubs and emphasized the value of providers offering transport.

All released individuals who spoke to service providers’ presence at transport hubs in their interviews expressed that this positively affected their release day experience. As one released individual expressed: *“It was a whole bunch of people [at the transportation hubs]. I don't know who them people was, but I thank God that they was there… I said, "Thank you so much." They said, "No, that's what we're here for. We're here to help you.” (R2195).* Another described: *“[Service providers] was there handing out food and water and stuff, and someone else was there with coffee and snacks. Great time, great day. I was getting released. I was happy. I couldn't wait to see my family.” (R2108).*

Providers reported several challenges they identified on the November 4^th^ release. One provider posited that, because many people were released before eating that day, released individuals were hungry and frustrated, which disinclined them to engage with reentry providers on drop-off. The provider described: *“Honestly at 11, 12 o'clock and they've been in line since six o'clock in the morning, they don't want to hear it. They don't want to talk, they're hungry.” (P183).* Another provider described observing released individuals discarding the envelopes containing release paperwork. Although this provider attributed individuals discarding their paperwork to impatience, it is possible that this behavior could be related to the theme detailed in “Pre-Release Services in Preparation for PHECA Release”: that some released persons did not understand the contents of their reentry plans. One provider reported that released persons’ families were given conflicting messages about where to meet loved ones, first having been told to meet at correctional facilities, then at transport hubs. *“Then the transit facilities started to get bogged down with family members that were told by the DOC to go there. And no one had information on the ground about where anyone was being transported to. So they're looking to us as providers to say, "Where's my loved one?" We have no idea. It got quite hectic and chaotic when we were there.” (P183).* In sum, all providers were in agreement that the November 4^th^ mass release was associated with various challenges, although each provider identified different specific areas of difficulty.

### Community services accessed after release

Many released person respondents expressed appreciation for the community reentry services that they were provided following release. Providers and released persons described that common services included housing and employment assistance; technology assistance; transportation; SUD treatment; assistance with benefit and ID applications; and linkages to other service providers. With this said, almost all interviewees, both released persons and service providers, reported reentry services were stressed by the dual challenges of mass release and pandemic precautions.

Specific to SUD treatment experiences: the nine interviewed individuals receiving MOUD pre-release reported varying experiences in their transition to care in the community. One respondent described being unaware that they could continue to receive MOUD services post-release. Some described challenges including dissatisfaction with prescription process (e.g., receiving fewer days-supply of medication than expected, difficulty redeeming prescriptions at pharmacy). All remaining individuals receiving pre-release MOUD reported smoothly transitioning to MOUD services in the community.

### Service system stress: after November 4^th^, during the pandemic

A theme observed in both reentry provider and released person transcripts was that, in the aftermath of the November 4^th^ mass release, community resources and services appeared overworked. *"It was difficult to keep up with. There was a lot of people that were released at one time and we're used to a few people leaving every day that are needing services. And so I think it kind of just shocked all systems.” (P170)* one provider said of their organization’s ability to meet released person needs following November 4^th^. Much as the quoted provider above, many interviewees described systemic barriers and backlogs as being attributable to many released persons simultaneously needing similar services. Another provider described; *“I think there were people who just got left out in the cold because there weren't beds places. There weren't slots in recovery programs. Recovery programs were backed up. I knew there were waiting lists, at a certain point.” (P192).* Respondents detailed that some of systemic backlog following the mass release was compounded by pandemic precautions limiting service enrollment capacities. Emergent themes related to pandemic precautions limiting services included provider and released person respondents reporting that services which did not previously have waitlists (e.g. local SUD treatment centers) needing to implement them; that there were fewer housing opportunities for unhoused individuals due to COVID-related holds on new residents; and that many local food banks were closed during the pandemic. Several released person respondents detailed that personal supports such as friends and family were a protective factor in managing these service gaps.

Amongst released person respondents, several individuals identified that a central challenge to service receipt was perceived disorganization within community service provider agencies. A commonly reported example of this disorganization was difficulty getting in touch with community agencies via phone. *“Any time I try to get a hold of [service provider] he's not there. I know he's multi-tasking. It's hard to pin him down and get him focused on what I got going. But I understand it's not all about me, he's got a lot of stuff.” (R2188)* one released person detailed. Several other releasee respondents described experiences where they arrived at agencies – at which they had been promised services or had completed phone screens – to find that agency staff did not expect them.

### J-CAP disruptions impacting reentry

One of the most commonly reported reentry challenges espoused by both provider and releasee respondents was that many PHECA-released individuals reentered the community without having been provided official state ID. Individuals released without official ID reported facing barriers including difficulty securing employment, housing, and doctor’s appointments. Although ID provision is intended to be standard for released individuals through J-CAP, this was one of several J-CAP procedures that released person respondents reported as not being completed as intended. One respondent spoke to their experience *“Before I left, they asked me to fill out the paper form on getting [my birth certificate] saying that it should be mailed to my house, to my address, but it never got mailed.” (R2117).* One provider posited that disruptions to benefit-provision were exacerbated by reentry service staff working remotely through COVID-19, stating *“A lot of that isn't anybody's fault, but an IT glitch… Have people working in offices more and you're not affected by COVID. You know the person that can override it in the system and different things like that.”(P183).*

Released person respondents described that it was valuable to receive benefit management support from reentry service providers (e.g., refiling for benefits, helping released persons activate community benefits). Several interviewees described that one NJ county jail sought to address this service-gap by offering to provide ID for any PHECA-released persons statewide.

### Remote service delivery

Many community services (e.g., reentry coordination services, SUD treatment/support groups) shifted delivery to remote due to pandemic precautions. Released person interviewees had mixed perspectives about remote services. Some respondents expressed appreciation for being able to access services remotely. Others described challenges associated with remote delivery: *“How are we telling someone that they need to log onto their telehealth appointment when they don't even have a phone?” (P183).* Amongst respondents who described barriers to remote service delivery, reported barriers included: released persons not having access to technology/internet; insufficient technology fluency; and people having relatively lower interest in engaging in telehealth services. 

Some community agencies and programming responded to technology-related barriers of this kind, and several released person and provider respondents described how agencies expanded services to provide in-office access to tablets for virtual meetings and appointments; to help released persons attain smartphones; and to provide didactic support to develop released persons’ technology fluency. One respondent detailed, having been provided a smartphone through participation in IRTS services: *"Because of that damn COVID, everything was being done over the phone. So that phone saved my life when I finally got it. Because you couldn't go and make appointments or nothing, I get Social Security disability, you couldn't go to welfare in person, you couldn't do nothing in person.” (R2154).* This was a perspective shared by all released persons who identified these means of addressing technology-related barriers, with interviewees appearing appreciative of these efforts.

### Respondents’ reflections and recommendations for future

Salient interview themes comprised provider and released person respondents’ perspectives on how to improve reentry to the community. Several provider and released person respondents expressed that the challenges observed in PHECA releases were not unique to large scale community reentry during COVID-19. One provider interviewee specifically identified the racial discrimination inherent to the criminal legal system as a factor in PHECA releases: *“The system is doing what it's supposed to do: continue oppressing individuals of color.” (P155).* Although no other interviewees were as explicit in naming racial discrimination, the theme of stigma was endorsed by other released person and provider interviewees. As one individual stated: *“Honestly, the toughest things with reintegration is still even people still being, I guess prejudiced would be the word.” (R2174).* Stigma as a barrier to care was referenced both directly and indirectly by participants. Many reported reentry challenges mapped onto those prevalent in literature that speaks to structural inequities in community reentry (Doyle et al., 2022; Harding et al., 2019; Stopka et al., 2022).

Following that all respondents whose interviews addressed learning about PHECA in advance reported challenges in gaining clarity on PHECA procedures, a common recommendation amongst these respondents was for increased information dissemination about release policy and procedures before PHECA’s implementation. Several provider respondents advocated that in future mass releases, systemic strain could be alleviated by increasing inter-organizational transparency with involved parties, and through efforts to include more community reentry service organizations in strategizing for the release. This can be observed in one provider’s suggestion: *“Correspond with community organizations, like, "We need your help." Hold a public meeting to bring all these people to the table. Because since we are your best assets so that we stop sending them in there [to carceral facilities] in the first place.” (P155).*

Another common theme amongst participants reflecting on the challenges associated with PHECA was that releases’ rapidity disrupted standard reentry procedures such as J-CAP. As one provider detailed: *“We went and rushed us through a whole process that was supposed to take six to nine months, in like a month.”(R2174).* Provider and released person respondents identified that there were not enough resources or time to effectively meet the scaled-up demand of large-scale releases. Reported resource deficits included insufficient funds, too few employees, and challenges in securing means of effective remote delivery. This contributed to problems with service delivery identified in study interviews, including: 1) insufficient time for correctional facilities to process/deliver government IDs; 2) issues with paperwork/benefits being filed correctly; and 3) released individuals struggling to connect with service providers during high-demand periods. A commonly identified solution to these challenges identified by provider respondents called for more resources to be allotted for needs assessment and service implementation in anticipation of mass release events.

## Discussion

This study reviewed the lived experiences of persons with SUDs released during PHECA and reentry providers serving them. Expert organizations recommended that decarceration efforts such as PHECA be enacted countrywide, as a means of mitigating COVID-19’s impact on incarcerated populations and addressing systemic inequities inherent to the criminal legal system (American Public Health Association, 2020; Wang et al., 2020). NJ’s implementation of PHECA legislation represented one of the largest-ever rapid reductions of a state prison population (Parmely, 2021; Sinha, 2021), and presented a crucial opportunity to examine how rapid mass decarceration during a public health emergency affects reentry for individuals with SUDs.

Interviews revealed a spectrum of themes, comprising shared experiences amongst released persons and idiosyncratic experiences specific to each person’s unique circumstances.

Both provider and released person respondents detailed barriers and challenges associated with PHECA releases. Frequently reported challenges included housing insecurity, food insecurity, difficulty accessing community services, insufficient employment opportunities, limited access to communication technology, and limited access to transportation. These are consistent with literature anticipating the needs of individuals returning to the community during the pandemic (National Academies of Sciences, Engineering, and Medicine, 2020; Howell et al., 2020; Desai et al., 2021) and the broader scholarship on reentry, which endorses that these challenges frequently present for individuals released from carceral facilities even during typical circumstances (Harding et al., 2019; Hlavka et al., 2015; Visher et al., 2017). Ultimately, this speaks to the under-resourced nature of the reentry system and provides impetus to continue strengthening present reentry services.

Themes also emerged that were pertinent to the unique circumstances of mass release during the COVID-19 pandemic. Many released individuals expressed frustration about navigating COVID-related changes to service provision, and providers corroborated that technology access and fluency issues impacted many released peoples’ access to reentry services. In response to these challenges, provider programs and organizations sought out novel adaptations (e.g., providing released persons with smartphones, providing technology fluency training to individuals, etc.), which released person respondents identified as meaningfully addressing these needs.

Many releasee and provider respondents also described that community reentry services appeared overburdened following the initial cohort of PHECA-releases, which was perhaps to be expected, given the unprecedented size of the release (Parmely, 2021; Sinha, 2021). This was observable in some organizations needing to implement waitlists – having not previously required them – and communication lapses between released persons and reentry service agencies. Despite these disruptions, released person respondents regularly shared appreciation for services accessed and individual service providers that communicated investment in their needs. Released person and provider respondents again spoke to how reentry service agencies flexibly approached emergent barriers to care (e.g., having prison social workers facilitate phone intakes with incarcerated individuals pre-release, conducting brief needs assessments at transportation hubs where released persons were dropped off) as a means of absorbing strain on the reentry service system.

Potentially attesting to the success of the reentry service system in addressing PHECA-released persons’ needs is that the preliminary quantitative data on PHECA release outcomes is promising. Analyses in January 2022 found that lower one-year recidivism rates for PHECA-released persons than past one-year recidivism rates in NJ (Yi, 2022a), and a study completed on the long-term outcomes of the November 4^th^ release cohort corroborated that re-arrest rates were similar to individuals released during typical circumstances (Yi, 2022b). Further investigation is merited to quantify PHECA-released persons’ rates of overdose, mortality, recidivism, and engagement with services and benefits over time.

PHECA legislature was enacted to mitigate the effects of COVID-19 on NJ’s incarcerated population and it was impactful in this effort, with rates of COVID-19 infection and mortality amongst incarcerated individuals and prison staff reducing significantly (New Jersey Department of Corrections, 2021). Although the function of PHECA was to address pandemic-related outcomes in NJ carceral facilities, this mass release also provides a meaningful opportunity to examine the calls for decarceration as a means of moving towards structural and racial equity (American Public Health Association, 2020; Kutateladze et al., 2014; Wang et al., 2020; Nowotny et al., 2021; Klein et al., 2021). NJDOC data on NJ’s carceral facility population has evidenced that individuals from all racial and ethnic groups were numerically reduced between January 2020 and January 2022, although the disproportionate representation of incarcerated Black individuals in the NJ carceral population remains stable (New Jersey Department of Corrections, 2020, 2022). Amongst this study’s respondents, racial inequity and stigma presented as an important theme that was seen as affecting individuals’ experiences through mass decarceration. This finding calls for further exploration as to how to use decarceration in service of racial equity, as both respondent experiences and NJ incarceration statistics support that decarceration in and of itself does not address systemic inequities.

### Limitations

This study’s evaluation of reentry successes and challenges was based on subjective respondent experiences. While subjective experiences yielded common themes, quantitative measures of reentry success are necessary for future study and analysis. Additionally, this sample provides valuable context as to the lived experiences of individuals with SUDs and service providers involved in PHECA releases. However, PHECA is a legislation that is both geographically and historically specific, so it is possible that themes may not effectively generalize beyond the scope of this study’s time and geography.

### Implications for future decarceration efforts

Interview themes revealed that the systemic challenges faced by individuals returning to the community during large scale release appeared similar to those faced during reentry under typical circumstances, and preliminary outcome data supports that large scale releases such as PHECA can be enacted without dramatically impacting the wellbeing of released individuals with SUDs or the communities to which they return (Yi, 2022a, 2022b). In preparing for future large scale releases, it is important for policymakers and public health officials to refer to the lived experiences of released persons and reentry service personnel, and attend to how large scale decarceration events can overburden an already under-resourced reentry system.

Given respondents’ identification that service system strain was amplified by resource deficits, it would benefit future large scale releases for additional resources to be allocated for reentry service providers to scale-up operations to meet increased demand. Savings in state expenses, made through reduced carceral occupancy, may provide budgetary opportunities for reinvestment in the reentry service system. With increased funding, pre- and post-release programming can be expanded/extended to maximize continuity of care between prison and the community.

Respondents frequently identified that standard release procedures (e.g. J-CAP, pre-release service access, etc.) were not carried out as efficiently as had previously been possible during typical circumstances. Individuals should be guaranteed prompt eligibility for Medicaid and social assistance benefits, should receive official identification, and ensured access to cell phones on release. Should release rapidity or a public health emergency disrupt released persons’ access to these necessities, it is important for policy and procedures to be quickly updated to meet the needs of individuals returning to their communities. Additionally, study themes emphasized the importance of ensuring released persons understand what services they are entitled to and how to access them.

Speaking to the specific needs of individuals with reported OUDs, it is crucial that released persons have pre- and post-release MOUD. Study respondents who accessed pre-release MOUD had variable experiences in accessing MOUD post-release, with some encountering more barriers than others, and all who encountered these barriers describing the experience as extremely disruptive. While many states are moving towards streamlining access to pre- and post-release MOUD for carceral populations, it is important that momentum is maintained for ongoing efforts to ensure access to these services, which radically reduce released persons’ negative outcome risks (Green et al., 2018; National Academy for State Health Policy, 2021).

Study respondents recommended that community stakeholders (e.g. reentry service organizations) should be incorporated into decarceration policymaking and planning to address challenges with large-scale release coordination. Correctional systems would benefit from beginning release preparation well in advance when possible, and coordinating closely with reentry service providers. This would allow for community organizations to be more effective in their implementation of successful reentry services (e.g. stationing staff at release drop-off locations).

With preparation and inter-organization collaboration, successful reentry to the community should be possible for individuals with SUDs who are released as part of large-scale decarceration. The successes and challenges identified by individuals involved in PHECA releases draw attention to areas where reentry services can be buttressed and expanded. These individuals’ lived experiences may inform future decarceration planning that can support released persons with complex reentry needs.

## Supplementary Information

**Additional file 1. **PHECA released participant interview guide.

**Additional file 2. **Reentry service provider interview guide.

## Data Availability

The datasets (i.e. interview transcripts) generated and analyzed in this study are not publicly available in efforts to ensure respondents’ privacy is maintained, given the personal nature of these interviews. Data can be made available by the corresponding author (MHB) on reasonable request.
