# Supplementary material for: Returning home during the pandemic: a thematic analysis describing experiences of people with substance use disorders released early from New Jersey prisons during COVID-19
Source: Health Justice. 2023 Feb 27;11:11. doi: 10.1186/s40352-023-00208-x (PMC9969013; doi:10.1186/s40352-023-00208-x)
Supplement: Supplementary file 1 — Additional file 1. PHECA released participant interview guide. [file 40352_2023_208_MOESM1_ESM.docx]

Supplemental Materials 1.0: PHECA Released Participant Interview Guide

*Interviewer Instructions: Throughout the interview guide, directions to the interviewer are enclosed in brackets and italicized. Questions to be asked are in regular font. Probes are shown as bullet points with italic text.*

*[Introduction]*

Thank you for agreeing to participate in this project. New Jersey’s recent law that allowed the early release of some prisoners during the coronavirus pandemic is one of the first in the nation, and we’re very curious about your experiences with reentry. We’re also interested in hearing about what challenges you’re facing and how service providers in the community could better assist you. We will give you a $50 gift card for taking the time to speak to us today. As a reminder, what you tell us is entirely confidential and we are prohibited from sharing information that can identify you, except as outlined in the consent form.

[*If participant consented to recording:* If it’s still OK with you, we would like to audio record this interview. The reason we record is that your feedback is very important to us and we don’t want to miss anything you say. In order to maintain your privacy, please avoid sharing your name or any other personal information during this interview. If you accidentally share this information and would like to remove it from recordings, please let us know.]

Do you have any questions before we begin?

*[Interview]*

1. To start, I’d like to ask you a few background questions about yourself:

- *Age:*
- *How would you describe your race/ethnicity:*
- *Prison released from:*
- *Release date:*
- *Time in prison:*

1. Next, I have some questions about your experiences with being incarcerated during a pandemic, which I can imagine is different than usual given the need to keep people apart. Were you incarcerated before the pandemic began (that is, before March 2020)?
2. What were some of the main changes to the daily activities and services provided in the prison that you experienced during the COVID-19 pandemic?
   - *Based on response to open-ended question, ask participant about how COVID-19 impacted the following:*
     1. *Access to services like drug treatment, health services, support groups, education, etc.*
     2. *Visits and communication with family and friends*
     3. *Recreation*
     4. *Legal services*
   - *What was it like being incarcerated during the pandemic and how did that compare to what it was like before the pandemic?*
   - *What impact did these changes have on your well-being?*
3. Please tell me about the types of services and programs you participated in in prison during the last year. Examples include drug treatment, mental health care, AA/NA, and vocational or educational programs.
   - - *How were the services/programs impacted by COVID-19?*
4. *[If respondent received drug treatment]* Did you receive medication-assisted treatment (MAT) while in prison? MAT includes Suboxone (buprenorphine), Vivitrol (naltrexone), and methadone.

*[If respondent states yes]* Please tell me more about your experiences with MAT in prison.

- - *What motivated you to begin MAT while you were incarcerated? Have you used MAT before?*
  - *Were you offered a choice of medications?*
  - *What were your reasons for beginning [buprenorphine/naltrexone] instead of [buprenorphine/naltrexone]?*
  - *What, if any, were your concerns about beginning MAT?*
  - *What dose of medication did you get and was it adequate?*
  - *Did you get your medication as a tablet, film, or injection?*
  - *What dose of medication did you get and was it adequate?*
  - *Do you plan to continue MAT now that you have been released?*
  - *Were prison staff supportive or unsupportive of your use of MAT?*

*[If respondent states no]* why not?

- - - *Were you offered MAT?*
    - *What (if any) were your concerns about taking MAT?*
    - *What kept you from taking MAT?*
    - *Are you on MAT now that you are home, or do you have plans to start?*

1. How did you prepare for your release? In other words, how did you get yourself ready for the transition from prison back to the community?
   - *Did you do it on your own or in communication with friends and family on the outside?*
   - *Did you get assistance from staff within the prison (e.g., case manager)?*
     1. *What did this entail?*
     2. *Did you have a reentry plan?*
     3. *What were the contents of the plan?*
     4. *How long before release did this planning process start?*
   - *Did you get assistance from staff/workers outside the prison (e.g., IRTS peer navigator)?*
     1. *What did this entail?*
     2. *Did you have a reentry plan?*
     3. *What were the contents of the plan?*
     4. *How long before release did this planning process start?*
2. How prepared did you feel for your release?
   - - *[Based on response, ask for more information (e.g., “what makes you say…”)]*
     - *How could you have been better prepared?*
     - *What would have helped you feel more prepared for release?*
3. Were any appointments in the community set up for you for after your release? For example, did you have appointments to see a doctor, go to drug treatment, or with social services?
   - *How were you informed of the appointments?*
   - *Did you have a document?*
4. Did you have Medicaid or another type of health insurance when you got out of prison?
   - *How did you know?*
   - *Did you get a card?*
   - *Did you have any problems accessing health care when you came home? If so, what were they?*
5. Did you have a place to stay when you got out of prison?
   - - *Where did you have to stay?*
     - *Is it permanent?*
     - *Did anyone help you arrange a place to live?*
     - *Are you satisfied with your living situation?*
6. How are you supporting yourself financially?
   - - *How did you support yourself financially when you first came home?*
     - *Do you have adequate financial support?*
     - *Are you getting any help with access to financial support or employment?*
7. What are the greatest challenges you face when it comes to your recovery and reentry?
8. What impact does the coronavirus (COVID-19) pandemic have on your recovery, reentry, or your overall wellness?
   - - *Has it been more difficult to stay connected to other people or develop a support network?*
     - *Has it had any impact on your ability to get a job, stable housing, access health care or drug treatment, or anything else you need?*
     - *Has it affected your mental health in any way, such as making you feel nervous or depressed?*
9. What are your main needs now that you are home? For example, are you in need of housing, employment, addiction treatment, medical care, legal assistance, etc.? For each of these needs:
   1. *How well did prison staff help you address them?*
      - *How could prison staff have better helped you address them?*
   2. *How much assistance are you getting with these needs now?*
      - *Who is providing this assistance?*
   3. *What would help you to better address these needs?*
      - *Are there certain programs or resources that would be helpful?*
10. That is all the questions I have for you. Thank you so much for your responses. Is there anything else you would like to add before we finish?
