# Supplementary material for: Returning home during the pandemic: a thematic analysis describing experiences of people with substance use disorders released early from New Jersey prisons during COVID-19
Source: Health Justice. 2023 Feb 27;11:11. doi: 10.1186/s40352-023-00208-x (PMC9969013; doi:10.1186/s40352-023-00208-x)
Supplement: Supplementary file 2 — Additional file 2. Reentry service provider interview guide. [file 40352_2023_208_MOESM2_ESM.docx]

Supplemental Materials 2.0: Reentry Service Provider Interview Guide

*Interviewer Instructions: Throughout the interview guide, directions to the interviewer are enclosed in brackets and italicized. Questions to be asked are in regular font. Probes are shown as bullet points with italic text.*

Do you have any questions before we begin?

*[Interview]*

1. Please describe how your organization prepared to support the large number of prisoners released in early November following passage of the Public Health Emergency Credit Act.

- *Was your organization adequately prepared?*
- *What else could your organization have done to better prepare?*

1. In what ways did the NJDOC and other state agencies coordinate with your organization to facilitate re-entry of prisoners released as part of Public Health Emergency Credit Act?

- *How did the NJDOC communicate with your organization to assist you in preparing for the release?*
- *How could coordination and communication be improved?*
- *How did coordination for this large-scale release compare to the ways the NJDOC coordinates with your organization to support released prisoners when large-scale releases are not going on?*

1. Were prisoners adequately prepared for their release by NJDOC?

- *In what ways were they inadequately prepared?*
- *How did preparation for those released due to the early release legislation compare to preparation for those released under ordinary circumstances (i.e., not released early)?*
- *What could have been done to better prepare prisoners for release?*

1. Are most people enrolled in Medicaid at the time of their release?
2. Are you finding that in most cases, released prisoners have identification documents at the time of their release?
3. Are prisoners being released with adequate supply of medication and appointments scheduled with medical and behavioral health care providers? Are there specific problems that you have identified in this area?
4. Please describe your experiences with linking released prisoners to needed services, including housing, health care, addiction treatment, and benefits.

- *How does your ability to link prisoners released as part of the NJ early release legislation to services compare to your ability to link prisoners released before the legislation?*
- *Have you identified any problems in the release process and the process of linkage to necessary follow-up services?*
- *What specific suggestions do you have for improving linkages to services like addiction and mental health treatment?*

1. What services do people need that they are not getting?

- *Which of these are most important?*
- *What are the reasons they are not getting the services? (e.g., services not available in the community, no identification, no insurance, lack of service provider capacity to make the linkage)*
- *What would need to change for people to get the services they need?*

1. What lessons have you learned in this process that you would want to share with reentry service providers preparing for large-scale prison releases?
